# The state of all-terrain vehicle (ATV)-related injury among Israeli children: Data and regulation

**DOI:** 10.3389/fpubh.2022.1029974

**Published:** 2022-12-20

**Authors:** Yigal Godler, Daniela Orr, Elad Calif

**Affiliations:** Beterem–Safe Kids Israel, Petah Tikva, Israel

**Keywords:** all-terrain vehicles (ATVs), children, socio-ecological model (SEM), falls, rollovers, regulation, Israel, Quad bikes

## Current situation

In the past, all-terrain vehicles (ATVs) were used in Israel exclusively for agricultural work, such that to purchase an ATV one had to offer proof that one worked in agriculture. This is no longer the case (1). The uses of these vehicles in Israel have expanded to include sports and leisure. According to researchers from the Gertner Institute for Epidemiology and Health Policy Research, approximately 680 ATVs or Quad bikes are sold annually in Israel, of which 12% are used for sports, 32% are used to train beginners, and 56% are used for leisure (1). Moreover, most of the injuries involving ATVs/Quad bikes in Israel occur in the context of leisure and sports activities, and not as part of agricultural work (1).

In what follows, we will inter alia offer data on how the level of ATV-related injury varies as a function of demographic variables, including ethnic background. Demographically, as of 2022, Israel's citizenry is comprised of 9.523 million people, of whom 7.069 million are of Jewish descent, 2.026 million were of Arab indigenous descent, and 498,000 identify as neither (2). In 2020, 3.049 million children (aged 0–17) lived in Israel, of whom 2.207 million were of Jewish descent (72.4%), and 737,000 were of Arab descent (24.2%) (3). An additional 105,000 children (3.4%) were classified as “others” (3) (see Box 1).

The legal and regulatory situation in Israel, as regards children's use of ATVs, is ambiguous. Most notably, because many ATVs are defined as toys under the law (4–6). On the one hand, Israeli laws prohibit adolescents younger than 16 from driving motorized ATVs (7) [articles 188 and 179 (8)]. These laws also require that the prospective driver possesses at least a minimal driving license (in Israel, a tractor license is considered to be the most basic for a motorized vehicle) (9). Moreover, Israeli law proscribes driving motorized ATVs in terrains defined as 'roads' by the Ministry of Transport and Road Safety, with the exception of roads enclosed in agricultural villages such as Kibbutzim (9). On the other hand, there are ambiguities in the laws pertaining to the use of electric ATVs by children and adolescents in Israel. The Standards Institution of Israel does not propose relevant standards for children's vehicles traveling at speeds exceeding 8 kph (kilometers per hour), including ATVs (4). Notwithstanding the noted regulatory ambiguity, the popularity of ATV usage is widespread in Israel (5). The number of ATVs in Israel is estimated in the thousands. According to news reports, 4,579 ATVs were sold in the year 2020 in Israel, 800 more than in 2019 (10)—these are dramatically higher figures than the approximate figures cited above, albeit for a slightly earlier period (1).

Three factors underscore the need for research into ATV-related injury in Israel: their popularity, the incidence of injuries, particularly among children, and the dearth of relevant data. According to Siman-Tov et al. [(9), p. 540], “[I]n Israel, crash mechanisms data are absent, as the National Trauma Registry does not record this information”. Nevertheless, data from around the world points toward the prevalence of falls, collisions and rollovers (9), as the leading crash mechanisms. Albeit exceeding the scope of the category of children, a retrospective study based on data from the Israeli National Trauma Registry, by Siman-Tov et al. [(9), p. 541] found that, between 2008 and 2016, “Non-Jews (31.5%), males (83.3%) and users 15–30 years old (64.5%) had the highest prevalence of ATV-related injuries,” with the category of non-Jews composed primarily of Arab ATV users.

Meanwhile, our data at *Beterem-Safe Kids Israel* (*Beterem*, in what follows), which focuses specifically on pediatric ATV-related injuries between 2012 and 2021, indicates that at least 267 children (defined as individuals aged 0–17) have incurred injuries from riding ATVs. At least in 112 of these cases (or 41%), the main cause of injury from riding ATVs were falls, in line with the extant research. Meanwhile, rollovers occurred in at least 43 of the cases (or 16%). More marginally, collisions while riding ATVs occurred in only 10 of the cases (that is, 3%). Judging by the data, the majority of cases involved motorized ATVs, although coding ambiguities preclude a clear-cut determination on the precise (motorized/electric) nature of the vehicle.

Box 1 **A statement on the use of ethnic categories in the text**

In what follows, we refer to two main ethnic groups which comprise Israeli society (though there are smaller ethnic groups in Israel as well): people of Jewish descent which constitute the ethnic majority and people of indigenous Arab (mostly Muslim) descent, who constitute an ethnic minority. As a shorthand, we will refer to the former as “Jews” and to the latter as “Arabs.” This is considered commonplace usage in Israeli society and in some of the scholarly literature cited herein.

## Current activities

Despite problematic regulation, several attempts to address the phenomenon of ATV-related injuries have been proposed in recent years. One policy that came to be implemented, was described in an internal document of the Israeli police, which was leaked to the news media (6). In this document, police officers were instructed to confiscate and destroy electric ATVs (6). In addition, they were required to open criminal investigations against riders failing to present driving licenses, registrations and insurances (6).

The said police document clarifies that electric ATVs are not vehicles under existing Israeli law. Nevertheless, insofar as ATVs are spotted on roads, police officers are instructed to treat them as vehicles (6). In other words, under the terms of the said police instructions, the same laws governing motorized vehicles are to be applied to electric ATVs, and, as noted above, said laws disallow motorized ATVs from traveling on roads. These laws also obligate drivers to carry driving licenses, vehicle registration documents and insurances. But since, as noted above, electric ATVs are not regarded as vehicles under the law, such documentation cannot be presented by definition.

From the perspective of Israeli law, an ATV would have to be defined as a vehicle in order to be licensed, registered and insured. The only such ATVs are motorized. However, the legal definition of motorized ATVs suffers from circular reasoning, as it defines vehicles by stipulation, ignoring most of their technical features. Indeed, the registration of ATVs as motorized ATVs is a precondition for conferring upon them the legal status of vehicles, no matter the technical commonalities an electric ATV might share with motorized ATVs (other than an engine) (6). To qualify as a motorized ATV, a vehicle has to be designed for exclusive use on unpaved roads and satisfy the following cumulative conditions (6):

1. it must possess at least four wheels;
2. the driver's sitting position must be such that the legs are on opposite sides of the seat;
3. it must be steered by handlebars;
4. it must be designed for the transportation of no more than two individuals including the driver;
5. its engine capacity must not exceed 1500 Cubic Centimeters;
6. it must be registered as a motorized ATV;

While electric ATVs satisfy most of these conditions (with the exception of Condition 5, as they do not possess an engine), they are not registered as motorized ATVs. For this reason, they are not regarded as vehicles.

Israel's National Road Safety Authority proposed in 2020 another measure, which may address the above legal ambiguities. It recommended the adoption of an OECD standard – a standard developed by the International Road Traffic Safety Data and Analysis Group (IRTAD). According to the proposed standard, the risk posed by a vehicle should be assessed according to four types: light and slow (Type A), light and fast (Type B), heavy and slow (Type C), and heavy and fast (Type D) (4, 8). Thus, the heaviest and fastest ATVs—those exceeding 25 kph and/or weighing over 35 kilograms (or types B-D)—would become legally equivalent to larger vehicles in the same speed and weight categories (4). However, so far, Israel has not implemented the proposed standard, though decision-makers may be moving in this direction.

## Current challenges

The noted legal and regulatory ambiguities which surround ATVs are not merely descriptive of the situation, but also constitute a challenge for those seeking to improve the state of child safety in Israel. However, there are other challenges. Such challenges operate at the institutional, cultural and individual levels.

For one, toy stores specializing in selling electric ATVs intentionally omit their top (that is, their maximal) speeds. Yet the ATVs sold in these stores may reach the speed of 70 kph. The said stores omit relevant information in order to avoid legal prosecution (4). They do so also in order to satisfy the customs classification of their ATV merchandise as ride-on toys. As it happens, this customs classification places the maximal speed of ride-on toy ATVs at 12.8 kph (4)—significantly below 70 kph, the actual speed of some of the ATVs sold.

In addition, and in line with the sociological characterization already cited from Siman-Tov et al. (9), children's ethnic background is likely to affect the probability of incurring ATV-related injuries. While the MDS form, used to gather the data comprising the MDS database, does not include direct questions on the child's ethnic background, it includes the municipality in which the child resides. Children residing in towns and cities wherein more than 80% of the population are Muslim-Arab, led to the imputation that the injured child's ethnic background was Arab. That is, because Arab majority towns in Israel are virtually homogenous from an ethnic point of view. But such imputation excludes ethnically mixed municipalities which are for the most part Jewish, with sizable Arab minorities. The methodological challenge of ethnically mixed cities also produced an underestimation of both Arab and Jewish children. Despite these limitations, our data indicate that in the years 2012–2021, Arab children incurred at least 70% of the injuries from being hit by ATVs as pedestrians, whereas Jewish children constituted at least 22% of those injured in this manner. At lower levels of injury incidence, Arab children constituted 29% of pediatric injuries from riding ATVs in the years 2012–2021. Broadening our scope to pediatric ATV-related injuries from both riding ATVs and being harmed by them otherwise (e.g., either as pedestrians or by various parts of the ATV while operating it), Arab children amounted to 32% of said injuries during the time-period under investigation. Meanwhile, Arab children constituted merely 24–26% of the overall population of children in the relevant years (correspondingly, Jewish children constituted 74–76% of Israel's population of children in the same years). These data indicate varying levels of Arab children's representation in the context ATV-related injury.

The slightly higher representation of Arab children than their share in the overall population, with reference to injuries resulting from riding ATVs, as well as their notable overrepresentation in being hit by ATVs, align with Beterem's (11) earlier findings regarding the overrepresentation of Arab children across other types of childhood injury. Similarly, the varying levels of representation of Arab children in ATV-related injuries reported herein (ranging from slightly higher than their share in the population to notably higher than their share), resonate with previous data on the overrepresentation of Israeli non-Jews from other age groups in ATV-related injuries. Although the category of “non-Jews” in Siman-Tov et al.'s (9) study, cited above, includes other minorities as well, Arabs constitute Israel's largest non-Jewish minority (~21% of the population).

Outside of the distribution of ethnic background, it is also worth mentioning that of the 267 children injured while riding ATVs, at least 191 (or 71%) were males, while only 75 (or 23%) were females (the rest are missing data). Moreover, approximately half of the ATV-related injuries for the studied time-period occurred among adolescents.

What the above data implies is a multilevel structure of sociological influences upon children's individual risk behaviors, in line with the socio-ecological model (SEM) (12, 13). As Baron-Epel and Ivancovsky [(12), p. 49] explain, the socio-ecological model is

[…] based on the idea that an interaction exists between the individual and the environment, where the individual's behavior is determined to a large extent by the physical and social environments. The behavioral, physical and social environments can be analyzed by the four ecological levels of society, from the individual level through the interpersonal level that includes family, friends and other social networks, through the community level that includes social norms, social institutions, and up to the societal level to include public policy on the national level.

They also point out that “[T]his model may serve as a good framework to understand the differences in injuries between a minority and majority population such as Arabs and Jews in Israel.” [(12), p. 49].

The derivative challenge is that preventive policy may be difficult to coordinate when the etiology of injury operates at multiple levels of social organization. That is, because modern society is a highly bureaucratic social setting, Israel being no exception. Moreover, besides the routine challenges of coordination, Israeli society is also plagued with various forms of institutional and budgetary discrimination vis-à-vis its Arab population (12). Indeed, previous cross-country research has established strong associations between, and proposed causal mechanisms relating, socioeconomic inequalities and a series of social and individual ills, including traffic accidents, and lack of adherence to safety measures as well as a tendency to dismiss other social norms (14).

## Future directions

We wish to echo the recommendation of Israel's National Road Safety Authority to adopt the OECD standard for the regulation of electric ATV use, and add a few others. For one, at the structural and regulatory levels, it is obvious that policy-makers must eliminate the gaps and ambiguities reviewed herein. Secondly, other institutions and their nature have to be taken into account. Thus, for instance, the profit motives of ATV dealers should be acknowledged and tackled. Provisions need to be put in place in order to prevent the withholding of adequate safety information from customers and regulators. For instance, fining perpetrators may prove helpful. Finally, in line with Siman-Tov et al. (9), we believe it is crucial to take into account the sociological characteristics of groups suffering from a higher incidence of injuries. These authors singled out the sociological categories of “non-Jewish, male and age group 15–29 years”, and emphasized the need to reckon with the “language and culture characteristics which should be considered in planning any public campaign” (p. 544). We wish to apply the same reasoning more neatly to pediatric ATV-related injuries. As we have seen, not only adolescents or males, but also Arab children, suffer from higher incidences of ATV-related injuries. The latter and their parents, require not merely culturally sensitive messaging, but also structural reforms addressing the economic and infrastructural strain on this underprivileged sector of Israeli society. Finally, in line with the foregoing, messages targeting Arab families must bear in mind the material limitations besetting their daily lives.

## Author contributions

YG was responsible for the write-up and data analysis. DO contributed to the conception of the study and to collecting literature. EC contributed to data extraction and analysis. All authors contributed to the article and approved the submitted version.
